# A Cohort Study Exploring HPV Vaccination Beliefs Among Oral Health Providers: Broadening the Scope of Education and Administration

**DOI:** 10.3390/vaccines12121331

**Published:** 2024-11-27

**Authors:** Leanne Brechtel, Larry C. Kilgore, Oluwafemifola Oyedeji, Alicia M. Mastronardi, Eric R. Carlson, Nikki B. Zite, Samantha Gregory, Jonathan Boone, Kristopher Kimball, Robert E. Heidel, Jill M. Maples

**Affiliations:** 1 Department of Obstetrics and Gynecology, College of Medicine, University of Tennessee Health Science Center, Knoxville, TN 37920, USAoluwafemifola.oyedeji@tennova.com (O.O.); ammastrondardi@utmck.edu (A.M.M.); nzite@utmck.edu (N.B.Z.); sgregory1@utmck.edu (S.G.);; 2 Department of Obstetrics and Gynecology, University of Iowa, Iowa City, IA 52242, USA; 3 Division of Gynecologic Oncology, Cancer Institute, University of Tennessee Medical Center, Knoxville, TN 37920, USA; 4 Internal Medicine, Tennova North Knoxville Medical Center, Knoxville, TN 37849, USA; 5 Department of Oral and Maxillofacial Surgery, College of Medicine, University of Tennessee Health Science Center, Knoxville, TN 37920, USA; 6 Department of Surgery, College of Medicine, University of Tennessee Health Science Center, Knoxville, TN 37920, USA; rheidel@utmck.edu

**Keywords:** human papillomavirus, dental health care, educational intervention

## Abstract

Background/Objectives: There is potential utility and increasing interest in engaging professionals in non-traditional vaccination settings to participate in efforts to reduce human papillomavirus (HPV)-related cancer. This study assessed the impact of a multi-disciplinary HPV educational intervention on oral health care professionals’ perceived role, comfort level, and scope of practice in HPV-related cancer prevention efforts. Methods: The virtual educational intervention was provided by a multi-disciplinary panel of experts. Seventy-three oral health care professionals attended the educational intervention and completed a questionnaire at three time points (pre-session, immediate post-session, and at the 1-month follow-up). Data were analyzed using Friedman’s ANOVA and post-hoc analyses. Results: Respondent’s median belief that it is the role of an oral health professional to recommend the HPV vaccine increased from pre-session (Median = 3.0, IQR = 3.0–4.0) to immediate post-session (median = 4.5, IQR = 4.0–5.0), and this increase was maintained 1 month after the session (median = 4.0, IQR = 4.0–4.5; *p* < 0.001). Additionally, respondent’s belief that they were up-to-date on the latest guidelines for HPV vaccination also increased from pre-session to immediate post-session (*p* < 0.05), and this increase was maintained 1 month after the session (pre-session median = 2.0, IQR = 2.0–3.0 vs. 1-month post-session median = 4.0, IQR = 4.0–5.0; *p* < 0.005). Conclusions: The multi-disciplinary HPV educational intervention was well-received by oral health professionals. Data suggest the intervention had a lasting impact on their beliefs about their role, comfort level, and scope of practice relating to HPV cancer prevention. More research needs to be conducted to better understand how obstetrician-gynecologists, other obstetric care providers, and oral health communities can support each other in promoting HPV-related cancer prevention.

## 1. Introduction

The human papillomavirus (HPV) is the most common sexually transmitted infection as well as the most common cause of newly diagnosed cervical cancer [1,2,3]. Oral squamous cell carcinoma (OSCC), which has been long associated with tobacco and alcohol use, has more recently been associated with HPV infection, and HPV infection has now become the most recognized cause of newly diagnosed oropharyngeal cancer (OPC) [3,4]. It is estimated that 70% of newly diagnosed oropharyngeal cancers are a result of the human papillomavirus, with associated risk factors including tobacco use, marijuana use, higher risk sexual behaviors (increased number of partners, oral sex, anal sex), and HIV diagnosis [5,6,7]. Recent studies have shown that among HPV strains, high-risk HPV types 16 and 18 can be attributed to approximately 62–90% of HPV-related oral cancers [2,8]. Despite medical advances, oropharyngeal cancer rates related to HPV have been increasing [3,9,10], with oncogenic HPV infection persistence being the leading cause of OPC. The number of squamous cell oropharyngeal rates increased from 1999 to 2015, with rates increasing among men by 2.7% and women by 0.8% [5].

The Centers for Disease Control and Prevention (CDC) has noted HPV vaccination efforts to be a public health priority given that HPV-associated cancers are currently the only known cancers that can be prevented with a vaccine. Despite this, vaccination uptake rates are suboptimal based on the 80% target set by Healthy People 2030 [11]. Nationally, only 62% of adolescents aged 13–17 years old completed the HPV vaccine series as of 2021 [12]. For adults aged 18–26 years old, only about 22% reported receiving the recommended doses of the HPV vaccine from data collected in 2018 [13].

Health care provider recommendation has been strongly associated with HPV vaccination adherence [14,15]. Given that most individuals in the United States reported at least one visit to dental offices a year [16] and that oral health care providers conduct oral cancer screening, oral health providers are uniquely positioned to provide HPV vaccine education and referrals with the goal of improving vaccination rates [17]. In 2018, the American Dental Association issued a policy that urged providers to support the use and administration of the HPV vaccine as a cancer prevention strategy [18]. While evidence suggests that oral health professionals are willing to discuss the HPV vaccine with their patients [19], few oral health care providers are discussing HPV-related cancers or are recommending the HPV vaccine in practice [20]. The most common reported barrier that prevents oral health care professionals from providing HPV vaccination education to their patients is a deficit in knowledge and training [19]. This perceived deficit often prohibits oral health providers from delivering an adequate message or recommendation [21]. Therefore, there is a need for training oral health care providers on the HPV virus, HPV-related cancers, the HPV vaccine, and how to provide an effective recommendation for HPV vaccination. More research is needed regarding increasing knowledge and confidence levels of oral health professionals [21]. As more is becoming known about the correlation between HPV and oropharyngeal cancers, the role oral health professionals could play in the education, promotion, and distribution of the HPV vaccine is currently being explored. The goal of this study was to assess oral health care providers’ knowledge of HPV and their perceived role, attitude, and comfort level in discussing the HPV vaccine before and after participating in an educational intervention.

## 2. Materials and Methods

### 2.1. Study Design, Setting, and Study Population

This prospective cohort study was conducted among oral health professionals affiliated with the Tennessee Dental Hygienists’ Association. Oral health care professionals were invited to attend a one-day online seminar session for continuing education, held on 5 March 2021, hosted by the Tennessee Dental Hygienists’ Association.

### 2.2. Description of Intervention and Implementation

A multi-disciplinary team of experts in head and neck cancers, gynecology oncology and HPV vaccine research was convened in early 2020. The team had regular meetings to discuss and finalize the content of the educational material based on current literature and Centers for Disease Control and Prevention (CDC) recommendations [22,23]. The educational material included the following topics: HPV infection, disease and epidemiology; survivor testimony; HPV-related oral and throat cancer (screening, diagnosis, and treatment); HPV vaccine (description, dosing and recommendation, impact, and safety); and how to provide an effective recommendation. Information was also provided on the available cancer prevention resources and toolkit, which has been described in prior studies [24]. Additional information describing the educational intervention is listed in Appendix B.

Participants were emailed a pre-seminar REDCap survey 2 days prior to the event. Participants then attended the virtual educational seminar delivered by the multi-disciplinary panel of experts. Immediately following the webinar, participants were emailed an individualized link to the post-webinar survey with the same questions as the pre-survey. Participants were asked if they would be willing to be contacted in 1 month for an additional follow-up survey and were asked if they would like to receive printed copies of an “HPV vaccination toolkit”. The HPV vaccination tool kit included publicly available HPV vaccine-related flyers, posters, and fact sheets developed by the American Cancer Society and American Academy of Pediatrics. The printed copies of the toolkit were mailed to participants who requested them after the webinar. All participants willing to be included received a 1-month post-webinar survey with the same questions as the pre-webinar and immediate post-webinar surveys. Participants received $40 as compensation if they completed all three surveys. This study was approved by the University of Tennessee Institutional Review Board (#4772). All participants provided consent to have their pre-, post-, and 1-month survey responses included in this study.

### 2.3. Survey Development, Administration, and Measures

Surveys were distributed to the participants electronically via REDCap pre-intervention, immediate post-intervention, and 1-month post-intervention. Survey questions were replicated and adapted from previously published questionnaires and administered to similar populations (7). This questionnaire included items that assessed the participant’s knowledge of HPV, their perceived role in HPV-related cancer prevention efforts, their comfort level in recommending the HPV vaccine, their current cancer screening practices, and demographic data. Survey questions used to measure perceptions and beliefs were in Likert scale format, with responses ranging from strongly disagree (1) to strongly agree (5). A list of survey questions and answer choices is in Appendix A.

The following five questions were asked to assess respondent knowledge of HPV and HPV vaccination at all three time points:

1. HPV is associated with the majority of oropharyngeal cancers in the United States.
2. HPV is associated with the majority of anal, cervical, and penile cancers.
3. HPV vaccine is recommended for men and women through age 26.
4. Most children who are 9 to 12 years old should get two shots of HPV vaccine six to twelve months apart.
5. HPV vaccination can clear an HPV infection that is already present.

Respondents were given the following answer choices: true, false, unsure. If respondents indicated they were unsure of the correct answer or incorrectly answered the question, they were assigned zero points. If they correctly answered the question, they were assigned 1 point. A composite knowledge score was calculated by adding the scores together from the five individual questions (ranging from 0 to 5) for each time point (pre-intervention, immediately post-intervention, and 1 month post-intervention).

### 2.4. Statistical Analysis

Descriptive statistics were used to describe the demographic and clinical characteristics of the sample. Pre-intervention measures of the participants’ perceived role in HPV cancer prevention were compared to responses immediately after the intervention and 1 month later using Freidman’s AVOVA. When a significant main effect was detected, post-hoc comparisons were performed using Wilcoxon pairwise tests. Data were collected using REDCap, and data analysis was performed using SPSS Version 29 (Armonk, NY, USA, IBM Corp.) Statistical significance was assumed at a two-sided alpha value of 0.05.

### 2.5. Reporting Guidelines

This study followed STROBE reporting guidelines for cohort studies. Additional information regarding the educational intervention in this study is found in Appendix B, which follows the TIDieR checklist and guide.

## 3. Results

### 3.1. Participant Characteristics

**Figure 1:**
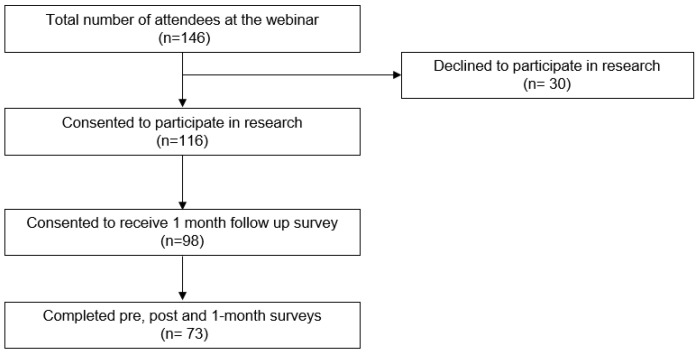
Study flow diagram.

A total of N = 146 oral health professionals attended the symposium. Of those, *n* = 30 declined to participate in research, while *n* = 116 (79.5%) consented to have their responses included in research efforts. Of the *n* = 116, *n* = 98 agreed to participate in follow-up, and *n* = 73 completed the 1-month post-intervention follow-up survey (74.5%). Those who did not consent to participate in the research project and did not complete all three surveys were excluded from the analysis. The final sample size used for analysis was *n* = 73 (Figure 1).

Of those included, 84.9% of respondents were dental hygienists, and 63.0% of respondents had been practicing for over 16 or more years (Table 1). The participants who indicated that they would like to receive the toolkit were provided with a copy (n = 73). However, only 60.3% (*n* = 44) indicated on the follow-up survey that they had received the toolkit. Of those 44 respondents, 93.2% (*n* = 41) reported the HPV vaccination toolkit was useful in their practice.

### 3.2. Participant Beliefs

There was a significant increase in participants’ beliefs measured across multiple survey items immediately after intervention, and many of these increases were sustained 1 month after the intervention (Table 2). There was a statistically significant and sustained increase in the respondent’s belief that discussing the link between HPV and oropharyngeal cancer falls within the scope of an oral health professional, with 82.2% (*n* = 60) of respondents strongly agreeing or agreeing prior to the intervention and 97.3% (*n* = 71) agreeing post-intervention (*p* < 0.001). This was sustained at 1 month, with 94.5% (*n* = 69) of participants again agreeing that this falls within their scope of practice (*p* = 0.175 from immediate post-intervention to 1 month post-intervention, indicating no change over the 1-month period). Only 1.4% (*n* = 1) of participants thought that administering the HPV vaccine in a dental office falls within the scope of an oral health professional prior to the intervention, while 53.4% (*n* = 39) of participants agreed or strongly agreed that it falls within the scope of an oral health professional following the intervention. This did fall to 38.4% (*n* = 28) at 1 month following the intervention. Similarly, 46.6% (*n* = 34) of participants believed it was their role to recommend the HPV vaccine to their patients pre-intervention, and this increased to 79.5% (*n* = 58) post-intervention and was sustained at 78.1% (*n* = 57) 1 month after. Two measures did not reach statistical significance: “My main concern with recommending the HPV vaccine is the time required to do so” (*p* = 0.099) and “Please rate your comfort level with performing an oral cancer screening” (*p* = 0.119; Table 2).

### 3.3. Participant Knowledge and HPV Recommending Practices

**Figure 2:**
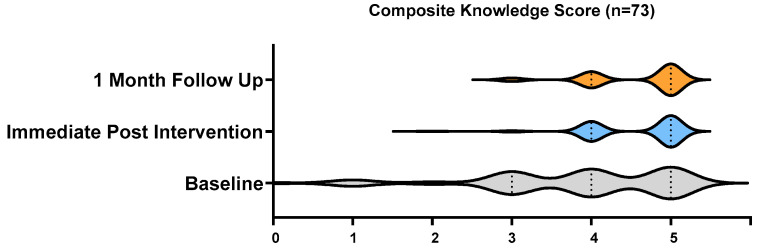
Composite knowledge score at baseline, immediate post-intervention, and 1 month post-intervention.

There was a significant (*p* < 0.001) improvement in the knowledge composite score from baseline to immediate post-intervention and no significant difference between the immediate post-intervention knowledge composite score and the one-month post-intervention composite score (Figure 2).

**Figure 3:**
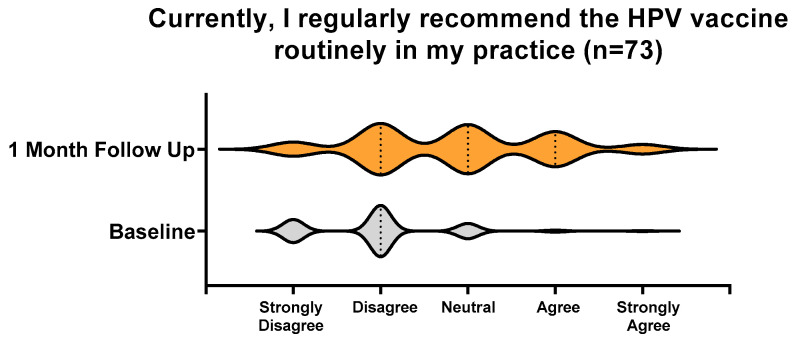
HPV vaccine recommendation practices at baseline and 1 month post-intervention.

A statistically significant increase in the number of respondents who currently regularly recommend the HPV vaccine in their practice increased from pre-intervention (n = 2 agree and n = 1 strongly agree; 3/73 = 4.1%) to the 1-month follow-up (n = 16 agree and n = 5 strongly agree; 21/73 = 28.8%; Figure 3).

## 4. Discussion

This study describes the effect of an educational intervention on oral health care providers’ knowledge of HPV and their perceived role, attitude, and comfort level in discussing the HPV vaccine. Our educational intervention demonstrated that oral health professionals gained an increased knowledge about HPV and its association with oral cancers and that this knowledge was sustained 1 month after the intervention (Figure 1). Additionally, there was a statistically significant increase in the opinion that discussing the link between HPV and oropharyngeal cancer falls within the scope of an oral health professional and that it is the role of an oral health professional to recommend the HPV vaccine to patients. This was consistent with previous studies demonstrating that oral health education initiatives are effective in preparing oral health professionals to discuss and recommend the HPV vaccine [24,25,26]. A notable tangible impact of our educational intervention was a statistically significant increase in respondents who stated they currently recommend the HPV vaccine in their practice from pre-intervention to the 1-month post-follow-up. It is important to recognize the unique role of oral health professionals regarding HPV awareness, prevention, and vaccination recommendations; thus, exploring multi-level factors that may influence routine HPV vaccine recommendations by oral health providers [17].

An interesting finding from this study is the non-significant increase in response to the question about time: “my main concern with recommending the HPV vaccine is the time required to do so”. In a different study by Shukla et al., the majority of oral health providers reported spending less than 5 min during clinical visits discussing HPV prevention [24]. In the same study, only a few (less than 10%) reported that time constraints were a barrier to providing the recommendation [24]. Other practice-related factors that may prevent the recommendation in a dental setting include the lack of a private space to have discussions and social or cultural norms [21].

The HPV vaccine has been FDA-approved and available for the prevention of HPV infections, including oncogenic strains that can lead to cancer. Over the past decade, many advances have been made within the field of obstetrics and gynecology, educating patients regarding the strong recommendation of receiving the HPV vaccine [18,19]. Currently, the American College of Obstetrician-Gynecologists recommends routine administration of the HPV vaccine to both males and females at a goal age of 11–12 years old [20]. With the increase in knowledge about the correlation between the human papillomavirus and oropharyngeal cancers, there is an opportunity to expand the promotion and distribution of the vaccine, and oral health professionals have been at the forefront of the current research. Expanding the network of health care providers could improve vaccination rates, screening practices, and overall patient education regarding the HPV vaccine and its association with cancer. The American Academy of Pediatric Dentistry has recognized that oral health care professionals are in an optimal position to provide education regarding HPV and the available vaccine. As adolescent patients visit oral health professionals more often than a medical doctor, there is an opportunity to build rapport and to provide repeated education over time [27]. Understanding the limitations of oral health professionals in recommending the HPV vaccine is of vital importance. This education session worked to begin the conversation and to offer a framework for education interventions in the future.

There were limitations within this study. This study was performed with a small sample size from a one-day intervention with a relatively homogeneous population. The main representation of this study population was dental hygienists, and there was not as strong a representation from advanced care providers or other positions within the dental office. Those who attended the educational session, consented to participate in this study, and completed all three surveys likely had a specific interest in this topic, and this may have contributed to selection bias. Notwithstanding, this study has several strengths. The study provided insights into knowledge and perceived role and attitude regarding HPV vaccination among oral health providers, primarily located in the Southeastern region of the United States of America. Therefore, these findings may not be generalizable to other health care providers or providers that are located in other regions of the world. However, collecting pre-, immediate, and one-month post-intervention data was a strength of the study, as this offered a broader scope of the impact of the educational intervention. The overall response rate for completing surveys at all three time points was 50.0% (n = 73/146) (Figure 1). There was a high response rate among those that agreed to participate in research and complete the 1-month follow-up survey, with 74.5% (n = 73/98; Figure 1) of respondents completing all three surveys.

Moving forward, more research needs to be done regarding how to better integrate HPV vaccine education, referral, and distribution within oral health education and practices. As the target age for vaccination is 9 to 12 years of age, this is an optimal time to encourage oral health professionals to incorporate a vaccine recommendation to their patients, as this age group often frequents oral health care facilities. Future HPV vaccination efforts should consider exploring opportunities to provide and distribute educational resources to oral health professionals, including how to promote HPV vaccine education, improve communication skills when recommending the HPV vaccine, and increase oral cancer screening.

## Figures and Tables

**Table 1:** Study participants’ characteristics (*n* = 73).

Characteristic	*n*	%
**Role**		
Dental assistant	5	6.8
Dental hygienist	62	84.9
Dentist	2	2.7
Other (dental hygiene educator, practice manager, retired, sales)	4	5.5
**Years in practice**		
0–2 years	3	4.1
3–5 years	2	2.7
6–11 years	8	11.0
11–15 years	9	12.3
16+	46	63.0
No longer practicing	3	4.1
Other	2	2.7
**Gender**		
Male	4	5.5
Female	67	91.8
Prefer not to answer	2	2.7

**Table 2:** Perceived role and comfort level regarding HPV cancer prevention (*n* = 73).

	Baseline (Median, IQR)	Immediate Post-Intervention (Median, IQR)	1-Month Post-Intervention (Median, IQR)	*p*-Value ^1^
Established professional policies exist regarding recommending the HPV vaccine to patients by oral health professionals.	3.0 (2.0–4.0)	4.0 (3.0–5.0) ^a^	3.0 (2.0–4.0) ^a,b^	<0.001
Discussing the link between HPV and oropharyngeal cancer falls within the scope of an oral health professional.	4.0 (4.0–5.0)	5.0 (4.0–5.0) ^a^	5.0 (4.0–5.0) ^a^	<0.001
Administering the HPV vaccine in a dental office falls within the scope of an oral health professional.	2.00 (1.0–3.0)	4.0 (3.0–4.0) ^a^	3.0 (2.0–4.0) ^a,b^	<0.001
In my dental training I adequately learned about HPV.	3.0 (2.0–4.0)	4.0 (2.0–4.0) ^a^	3.0 (2.0–4.0)	<0.037
In my dental training I adequately learned about the HPV vaccine.	2.0 (2.0–3.0)	3.0 (2.0–4.0) ^a^	3.0 (2.0–4.0) ^a^	0.001
I believe it is my role as an oral health professional to recommend the HPV vaccine to my patients.	3.0 (3.0–4.0)	4.5 (4.0–5.0) ^a^	4.0 (4.0–5.0) ^a^	<0.001
I have the training to effectively recommend the HPV vaccine to the correct patient populations.	2.0 (2.0–3.0)	4.0 (4.0–5.0) ^a^	4.0 (4.0–4.0) ^a,b^	<0.001
I trust the safety and efficacy of the HPV vaccine.	4.0 (3.0–4.0)	4.0 (4.0–5.0) ^a^	4.0 (4.0–5.0) ^a^	<0.001
I am up-to-date on the latest guidelines for HPV vaccination.	2.0 (2.0–3.0)	4.0 (4.0–5.0) ^a^	4.0 (4.0–5.0) ^a^	<0.001
My main concern with recommending the HPV vaccine is the time required to do so.	3.0 (2.0–3.0)	2.0 (2.0–4.0)	3.0 (2.0–4.0)	0.099
I am comfortable discussing my patient’s sexual history with them.	3.0 (2.0–3.0)	3.0 (2.0–4.0) ^a^	3.0 (2.0–3.5) ^a^	0.002
Please rate your comfort level with performing an oral cancer screening.	4.0 (3.0–4.0)	4.0 (3.0–5.0)	4.0 (3.0–5.0)	0.119

^1^ *p*-value for Friedman’s ANOVA statistical test. ^a^ significant change from baseline (*p* < 0.05, Wilcoxon post-hoc analysis), ^b^ significant change from immediate post-intervention to 1-month post-intervention (*p* < 0.05, Wilcoxon post-hoc analysis). Data are presented as median (IQR) where IQR is the interquartile range between 25th and 75th percentile.

## Data Availability

The datasets generated during and/or analyzed during the current study are not publicly available but are available from the corresponding author on reasonable request.

## Appendix A

### Survey Questions

(1) HPV is associated with the majority of oropharyngeal cancers in the United States.	True False Unsure
(2) HPV is associated with the majority of anal, cervical, and penile cancers.	True False Unsure
(3) HPV vaccine is recommended for men and women through age 26.	True False Unsure
(4) Most children who are 9 to 12 years old should get two shots of HPV vaccine six to twelve months apart.	True False Unsure
(5) HPV vaccination can clear an HPV infection that is already present.	True False Unsure

**Please Indicate Your Response for Each Statement About Oral Health and HPV**							
	**Strongly Disagree**		**Disagree**	**Neutral**	**Agree**	**Strongly Agree**	
Established professional policies exist regarding recommending the HPV vaccine to patients by oral health professionals.							
Discussing the link between HPV and oropharyngeal cancer falls within the scope of an oral health professional.							
Administering the HPV vaccine in a dental office falls within the scope of an oral health professional.							
Please Indicate Your Response for Each Statement About Oral Health and HPV							
		Strongly Disagree	Disagree	Neutral	Agree	Strongly Agree	Not Applicable
In my dental training I adequately learned about the human papillomavirus (HPV).							
In my dental training I adequately learned about the HPV vaccine.							
I believe it is my role as an oral health professional to recommend the HPV vaccine to my patients.							
Please indicate your response for each statement about concerns with recommending/administering the HPV vaccine.							
			Strongly Disagree	Disagree	Neutral	Agree	Strongly Agree
I have the training to effectively recommend the HPV vaccine to the correct patient populations.							
I trust the safety and efficacy of the HPV vaccine.							
I am up-to-date on the latest guidelines for HPV vaccination.							
Please indicate your response for each statement about concerns with recommending/administering the HPV vaccine.							
		Strongly Disagree	Disagree	Neutral	Agree	Strongly Agree	Not Applicable
My main concern with recommending the HPV vaccine is the time required to do so.							
I am comfortable discussing my patient’s sexual history with them.							
Currently, I regularly recommend the HPV routinely in my practice.							

Have you ever received training to perform an oral cancer screening?	Yes No Unsure Other: _____________
If you work in (or are being trained in) a dental office setting, does your office have a written policy on screening for oral cancer?	Yes No Unsure Not applicable – I am not working in or training in a dental office setting. Other: ______________
Do you perform oral cancer screenings?	Yes No I currently do not, but I used to perform oral cancer screenings. Other: _______________
What is your age?	
With which gender do you most identify?	Male Female Non-binary Prefer to self describe Prefer not to answer
What is your ethnicity?	Hispanic/Latino Non-Hispanic/Latino Prefer not to answer
What is your race?	White Black Asian/Pacific Islander Native American/Alaskan Native Multiple Prefer not to answer Other: _________
What is your current role?	Dental Assistant Dental Hygienist Dental Lab Technician Dentist Other: _________
How many years have you been practicing?	0–5 years 6–10 years 11–15 years 16+ years No longer practicing Other: _________

## Appendix B

Additional information regarding the educational intervention in this study following the TIDieR checklist and guide.

**The TIDieR (Template for Intervention Description and Replication) Checklist:**	
**BRIEF NAME**	
Provide the name or a phrase that describes the intervention.	Zoom Webinar titled: “HPV, Cancer, and Dental Health Providers Symposium”.
**WHY**	
Describe any rationale, theory, or goal of the elements essential to the intervention.	A multi-disciplinary panel of experts presented relevant HPV research and epidemiology, provided an overview of HPV-associated cancers and prevention strategies, and also discussed cancer screening, diagnosis and treatment, and HPV vaccinations. The goal of the seminar was to increase knowledge and awareness of HPV-associated oral cancer and HPV-attributed oropharyngeal cancers and to equip oral health care professionals with increased knowledge of HPV and tools for providing an effective HPV vaccine recommendation.
**WHAT**	
Materials: Describe any physical or informational materials used in the intervention, including those provided to participants or used in intervention delivery or in the training of intervention providers. Provide information on where the materials can be accessed (e.g., online appendix, URL).	The virtual educational intervention included seven presentations presented by eight different speakers. The format was primarily didactic; however, following each presentation, 5–10 min was built into the programming to allow for questions and discussion. The content of the educational material was based on current literature and Centers for Disease Control and Prevention (CDC) recommendations. All speakers presented a slide deck. These materials have not been shared publicly, but reasonable requests, made directly to the presenters, may be accommodated.
Procedures: Describe each of the procedures, activities, and/or processes used in the intervention, including any enabling or support activities.	The virtual educational was delivered in a single day from 8 a.m. to 2 p.m. (6 h total) and offered six continuing education credits to attendees. The event was hosted by the Tennessee Dental Hygienists’ Association on 5 March 2021. The online platform used to deliver the education was Zoom. The education was divided into seven distinct presentations (described in greater detail below) with opportunities for question and answer sessions following each presentation.
**WHO PROVIDED**	
For each category of intervention provider (e.g., psychologist, nursing assistant), describe their expertise, background, and any specific training given.	The education was provided by a team of experts or those with lived experience. “Expert” in this context is defined as an individual with specific knowledge of some aspect of HPV. All of the individuals had prior lived experience, clinical experience, and/or research experience related to HPV. The following in a list of speakers, their credentials and the title and brief description of their presentations: (1) Jill Maples, PhD. Dr. Maples was a co-investigator on a research grant funded by the University of Tennessee Cancer Institute focused on reducing the incidence of HPV-related cancers. She was a member of Immunize TN and part of a collaboration for state comprehensive cancer programs and coalitions (the Tri-Networks HPV Vaccination Learning Collaborative). She presented “Cancer Prevention Research Efforts”. This session provided a background on efforts bringing providers from different specialties and backgrounds together to eliminate HPV-related cancers. Joint statements from professional organizations supporting these efforts were presented along with ongoing research in this area in the state of Tennessee. (2) Larry Kilgore, MD and Gynecologic Oncologist. Dr. Kilgore has over 30 years of experience in Gynecologic Oncology, serving as an academic clinician and senior scientist at the University of Alabama at Birmingham and the University of Tennessee Medical Center at Knoxville. He has an extensive track record of HPV-related cancer research publications and experience, and is a passionate advocate for patients diagnosed with cancer. He presented “HPV Infection, Disease, and Epidemiology”. This session provided an overview of the epidemiology and disease association of HPV infections. The papillomaviruses were reviewed, an overview of how these viruses are spread was provided, and the abnormal tissue growth and cellular changes these viruses cause were presented, and morbidity and mortality of these processes were presented. (3) Tinisha Key. She is a cancer survivor. She presented “Survivor’s Testimony”. Tinisha’s personal battle was shared. (4) Eric Carlson, DMD, MD, EdM, FACS. With over 150 papers, 60 book chapters, and 5 books published in the field, Dr. Carlson is a leader in translational research initiative focused on the identification of human papillomavirus in oral and oropharyngeal cancer and the preoperative staging of head and neck cancers. He presented “HPV-related Oral and Throat Cancer: Screening, Diagnosis, and Treatment”. This session provided an overview of the prevalence of oral and throat cancer incidence and discussed human papillomavirus as an etiologic factor for oral and oropharyngeal cancers. The role of the dental health provider in screening for oral and throat cancers was covered. Diagnosis and treatment of these cancers were also presented. (5) Courtney Riedinger, MD, and Brandon Hays, MD. Dr. Riedinger was in her fourth year of OBGYN residency training, pursuing fellowship training in Gynecologic Oncology. Dr. Hays was also an OBGYN resident with a background in public health. They presented “Vaccine: Description, Dosing, and Recommendation, Impact, and Safety”. The session provided the most up-to-date information on the impact and safety of the HPV vaccine. Current CDC Advisory Committee on Immunization Practices (ACIP) guidelines for the HPV vaccine were also covered during this session. (6) Tiffany Skinner, DNP, RN, APRN, APN-BC. Dr. Skinner has an extensive background in implementing provider education models throughout the East Tennessee region. She has experience delivering vaccine-related education to and collaborating with providers in a variety of settings and across multiple specialties. Her work focuses on assessing barriers to vaccine uptake and increasing vaccine access and coverage. She is a leader in HPV vaccination recommendation strategies. She presented “Providing an Effective Recommendation”. This session provided evidence-based strategies for providing effective preventative health recommendations to patients, specifically focusing on HPV vaccination efforts. (7) Amy Fields, BA. Amy served East Tennessee as the Cancer Control Strategic Partnerships Manager for the American Cancer Society, partnering with hospital systems to make an impact on cancer prevention and early detection and quality of life for cancer patients and caregivers. She presented “Cancer Prevention Resources and Toolkit”. This session provided a brief overview of HPV-related cancer prevention efforts currently supported by major non-profit organizations and the US Department of Health and Human Services. Resources and materials, such as posters and informational brochures that can be used to enhance cancer prevention efforts, were also shared.
**HOW**	
Describe the modes of delivery (e.g., face-to-face or by some other mechanism, such as internet or telephone) of the intervention and whether it was provided individually or in a group.	The mode of delivery was virtual (internet). The education was delivered in a group format.
**WHERE**	
Describe the type(s) of location(s) where the intervention occurred, including any necessary infrastructure or relevant features.	The speakers included in the education were located in Knoxville, TN. The education was delivered to oral health care providers affiliated with the Tennessee Dental Hygienists’ Association.
**WHEN and HOW MUCH**	
Describe the number of times the intervention was delivered and over what period of time including the number of sessions, their schedule, and their duration, intensity or dose.	The intervention was delivered one time on 5 March 2021.
**TAILORING**	
If the intervention was planned to be personalized, titrated or adapted, then describe what, why, when, and how.	The intervention was planned to be personalized to oral health care providers practicing in the state of Tennessee.
**MODIFICATIONS**	
If the intervention was modified during the course of the study, describe the changes (what, why, when, and how).	The intervention was not modified.
**HOW WELL**	
Planned: If intervention adherence or fidelity was assessed, describe how and by whom, and if any strategies were used to maintain or improve fidelity, describe them.	The intervention adherence or fidelity was not assessed.
Actual: If intervention adherence or fidelity was assessed, describe the extent to which the intervention was delivered as planned.	Not applicable.
Hoffmann T, Glasziou P, Boutron I, Milne R, Perera R, Moher D, Altman D, Barbour V, Macdonald H, Johnston M, Lamb S, Dixon-Woods M, McCulloch P, Wyatt J, Chan A, Michie S. Better reporting of interventions: template for intervention description and replication (TIDieR) checklist and guide. BMJ. 2014;348:g1687.	
